# Suppression of *PtrDUF579-3* Expression Causes Structural Changes of the Glucuronoxylan in *Populus*

**DOI:** 10.3389/fpls.2016.00493

**Published:** 2016-04-11

**Authors:** Dongliang Song, Jinshan Gui, Chenchen Liu, Jiayan Sun, Laigeng Li

**Affiliations:** National Key Laboratory of Plant Molecular Genetics, Center for Excellence in Molecular Plant Sciences, Institute of Plant Physiology and Ecology – Chinese Academy of SciencesShanghai, China

**Keywords:** DUF579, glucuronoxylan, glucuronic acid methyltransferases, wood engineering, *Populus*

## Abstract

*DUF579* (domain unknown function 579) genes have been reported to play diverse roles in cell wall biosynthesis, such as in glucuronoxylan (GX) synthesis. As GX is a major type of hemicelluloses in hard wood species, how *DUF579* genes function in wood formation remains to be demonstrated *in planta*. This study reports a *Populus DUF579* gene, *PtrDUF579-3*, which is characterized for its function in wood cell wall formation. *PtrDUF579-3* is localized in Golgi apparatus and catalyzes methylation of the glucuronic acid (GlcA) in GX biosynthesis. Suppression of *PtrDUF579-3* expression in *Populus* caused a reduction in both the GlcA side chain number and GlcA side chain methylation on the GX backbone. The modified GX polymer through *PtrDUF579-3* suppression was more susceptible to acid treatment and the *PtrDUF579-3* suppressed plants displayed enhanced cellulose digestibility. These results suggest that *PtrDUF579-3* is involved in GX biosynthesis and GX structure can be modified through *PtrDUF579-3* suppression in *Populus*.

## Introduction

Being an abundant hemicellulose in wood, glucuronoxylan (GX) interacts with cellulose microfibrils and lignin via various connections and such interaction can contribute to recalcitrance of the conversion of lignocellulosic wood biomass into biofuel (Himmel et al., 2007). The GX in woody and herbaceous dicot plants has a backbone of β-1,4-xylosyl (Xyl) chain and there is a structure of the β-D-Xyl*p*-(1→3)-α-L-Rha*p*-(1→2)-α-D-Gal*p*A-(1→4)-D-Xyl*p* tetrasaccharide at its reducing end (Johansson and Samuelson, 1977; Lee et al., 2012a). In *Arabidopsis*, the GX backbone is usually substituted at C-2 with both α-glucuronic acid (GlcA) and 4-*O*-methyl α-glucuronic acid (4-*O*-MeGlcA), while such substitution on the GX backbone predominantly consists of 4-*O*-MeGlcA in *Populus* (Timell, 1967; Lee et al., 2011). In *Arabidopsis*, GX is synthesized through several glycosyltransferases (GTs), such as GT43 (IRX9, IRX14), GT47 (IRX10/GUT2, IRX10-L/GUT1, IRX7/FRA8, F8H), and GT8 (IRX8/GAUT12, PARVUS/GLZ1, GUX1/2/3) family proteins. These GT proteins are involved in different stage during GX biosynthesis processes. A set of GT proteins IRX9, IRX14, IRX10 and IRX10-L are required for elongation of the β(1-4)-xylan backbone, while other GT proteins IRX7, IRX8, and PARVUS are involved in synthesizing tetrasaccharide at the reducing end (Zhong et al., 2005; Bauer et al., 2006; Brown et al., 2007, 2009; Lee et al., 2007a,b, 2009b; Wu et al., 2009, 2010). Mutation of these genes decrease GX content, meanwhile mutation of *IRX7, IRX8*, and *PARVUS-3* leads to a reduction of the reducing end tetrasaccharide. The addition of GlcA residues onto xylan backbone is reported to be mediated by GT8 family protein GUX1/2/3 (Mortimer et al., 2010; Lee et al., 2012a). Mutation of *Arabidopsis GUX1/2/3* genes leads to absence of GlcA branches. In *Populus*, GT43A/B and GT43C/D are reported to be the orthologs of *Arabidopsis* IRX9 and IRX14, respectively, and poplar GT47C, GT8D, and GT8E/F are reported to be the orthologs of *Arabidopsis* IRX7/FRA8, IRX8, and PARVUS, respectively. The function of these proteins in GX biosynthesis was reported to be conserved in *Populus* compared to their *Arabidopsis* orthologs (Zhou et al., 2006, 2007; Lee et al., 2009a,c, 2011; Li et al., 2011).

In addition to GX backbone synthesis, GX is typically modified with GlcA side chains which appears to be methylated or not. In *Arabidopsis*, methylation of the GlcA side chain in GX was found to be mediated by DUF579 family proteins. Double mutant of DUF579 genes *irx15* and *irx15l* show an increase of GX methylation and decrease of GX content, which causes secondary cell wall defects (Brown et al., 2011; Jensen et al., 2011). Whereas, some other studies showed that DUF579 proteins have a catalytic activity to methylate GlcA residue in GX and mutation of the *DUF579* genes led to reduction of GX methylation (Lee et al., 2012b; Urbanowicz et al., 2012). It is unclear whether these different *DUF579* genes have different catalytic activities in the GX biosynthesis.

*DUF579* gene family consists of 10 members in *Arabidopsis* and 12 members in *Populus*. Our study suggests *Populus DUF579* genes play diverse roles in affecting wood formation in *Populus* (Song et al., 2014). They display a variety of expression patterns across various tissues and their encoded proteins are localized in different types of cells. *PtrDUF579-1* suppression specifically affected cambium cell wall formation (Song et al., 2014), while *PtrDUF579-3* and *PtrDUF579-4* are specifically expressed in xylem cells. In another study, *PtrDUF579-3* and *PtrDUF579-4* are reported to catalyze the GlcA methylation of GX *in vitro* (Yuan et al., 2014). However, it remains to be demonstrated how these *DUF579* genes function in regulating GX methylation *in planta*. Here we show that regulation of *PtrDUF579-3* expression resulted in GX structure modification in *Populus*. This study provides a line of new evidence for elucidation of the *PtrDUF579-3* function in GX biosynthesis during wood formation.

## Materials and Methods

### Plant Materials and Growth Condition

*Populus trichocarpa* was used for gene cloning. *Populus × euramericana* cv. *‘Nanlin895’* was used for genetic transformation analysis. Young *Populus* plants were grown in a phytotron with a light and dark cycle of 16 and 8 h at 22°C. *Populus* trees at 3-months were grown in a greenhouse as described (Song et al., 2014).

### Gene Constructs and Genetic Transformation

Total RNA was extracted from developing xylem using modified CTAB method (Chang et al., 1993). The total RNA used for gene cloning and gene expression analysis was extracted from developing xylem tissues of WT or *anti-PtrDUF579-3* transgenic plants. After removing DNA contamination, 1 μg of total RNA was reversely transcribed into cDNAs using HiScript II 1st Strand cDNA Synthesis Kit (R211, Vazyme, Nanjing, China). The full coding sequences of *PtrDUF579-3* and *PtrDUF579-4* were amplified and cloned from cDNA of *P. trichocarpa*. To construct antisense vector, the coding sequences of *PtrDUF579-3* was reversely inserted into pBI121 binary vector under a constitutive *CaMV35S* promoter. The constructs were transferred into *Populus × euramericana* cv. *‘Nanlin895’* by *Agrobacterium* mediated transformation according to the protocol adopted in our lab (Li et al., 2003).

### Subcellular Localization and Colocalization Experiment

*PtrDUF579-3* and *PtrDUF579-4* gene cloned above were fused with *mCherry* gene and inserted into pm-rk binary vector after removing *PIP2A* gene, respectively (Nelson et al., 2007; primer sequences for constructs supplied in Supplementary Table S1). The constructs of G-gk (Nelson et al., 2007) were used for Golgi identification. The two constructs and G-gk were then transformed into *Agrobacterium*, co-injected into tobacco leaves and were observed under laser confocal scanning microscopy (LCSM; LSM510 META, ZEISS, Germany) according to previous procedure (Song et al., 2014).

### Gene Expression Analysis

Gene-specific primers were designed to amplify a specific fragment (100–300 bp in length) of the *PtrDUF579-3, PtrDUF579-4*, and *PtrDUF579-9* genes (Supplementary Table S1). Using the cDNA prepared above as template, quantitative real-time PCR was performed using Hieff^TM^ qPCR SYBR^®^ Green Master Mix (11201ES03, Yeasen, Shanghai, China) on a MyiQ Real-Time PCR Detection System (Bio-Rad, Winston-Salem, NC, USA). The gene expression was normalized against *PtrActin2* expression and analyzed by Delta CT method using three biological replicates from independent plants.

### Microscopy Analysis of *Populus* Secondary Growth

For light microscopy analysis, the 12th internode of *Populus* stems were cut into 2 mm lengths and fixed by FAA solution (5% formaldehyde, 10% acetic acid, and 50% ethanol), dehydrated with ethanol and embedded in paraffin. After obtaining 10-μm sections with a Leica RM2235 rotary microtome and removing of paraffin, sections were stained with 0.05% toluidine blue and analyzed using an OLYMPUS BX51 light microscope according to previous protocol (Song et al., 2014).

### Measurement of Mechanical Strength

Mechanical strength was measured using a material tester (Instron 5867, Norwood, MA, USA). The mechanical strength was measured as the force required for breaking a fresh stem segment with a diameter about 1 cm and length about 10 cm. The mechanical strength was assayed using Young’s modulus, Y = (F/S)/(ΔL/L) (Mishra et al., 2000). Y, Young’s modulus; F, force; S, cross-sectional area; ΔL, changes of the stem in length; L, the original length of the stem.

### Chemical Analysis of Secondary Cell Wall Components

Wood developing xylem tissues (5–6 g) collected from stems of 1-year-old trees were ground in liquid nitrogen into a fine powder to prepare alcohol insoluble residues (AIRs) according to (Foster et al., 2010). After de-starched procedure, the cell walls AIR were subjected to determination of the chemical composition. The AIRs were hydrolyzed with 2 M trifloroacetic acid (TFA) at 120°C for 1.5 h. The produced monosaccharides were then dried in a rotatory evaporator and subjected to mercaptalation and silylation as described (Lluveras-Tenorio et al., 2012; Song et al., 2014). The final reaction mixture was dried with nitrogen flow, reconstituted in hexane and then were subjected to GC–MS analysis. The sugars and uronic acids were separated by HP-5 column, and detected by Agilent 5975 inert MSD system (Agilent, Santa Clara, CA, USA). Crystalline cellulose content and monosaccharide composition was analyzed according to the protocol previously described (Li et al., 2009). Lignin content was determined by Klason method and lignin subunit S/G-ratio was analyzed by thioacidolysis according to our previous protocol (Gui et al., 2011).

### GX Oligosaccharide Preparation

Above prepared AIR (1 g) was sequentially extracted with 50 mM ammonium oxalate and 1 M KOH, containing 1.0% (w/v) sodium borohydride. The KOH extracts were neutralized, dialyzed, and lyophilized. GX oligosaccharides were generated by digestion of the lyophilized KOH extracts (20 mg) with a *Trichoderma viride* endoxylanase (Megazyme) in 0.1 M ammonium formate buffer (pH 5.0) for 24 h at 37°C as described (Mazumder et al., 2012). The reaction mixture was neutralized with ammonia and passed through a cut-off ultrafiltration tube (3 KD, Millipore) to remove residual polysaccharides. Then the percolate was re-purified by adding ethanol to a final concentration of 65% (vol/vol) and concentrated to remove precipitates. The soluble fraction was subjected to liquid chromatography-quadrupole-time-of-flight-mass spectrometry (LC-QTOF-MS) analysis. GX oligosaccharides (about 25–30 mg) generated from digestion of 100 mg KOH extracts were desalted and lyophilized for ^1^H NMR analysis.

### GX Glucuronosyltransferase and Methyltransferase Assay

Microsomal proteins from developing xylem tissue were prepared according to previous method (Suzuki et al., 2006). The microsomes were washed three time with ice cold Milli-Q water and dissolved in protein extraction buffer containing 100 mM NaAc, pH 7.0, 0.5% w/v dodecyl-β-D-maltoside (DDM) and 1 mM protease inhibitor cocktail (Sigma). After centrifuged at 10000 *g* for 10 min at 4°C, the supernatant containing microsomal protein was collected and subjected to protein concentrations determination using Bio-Rad protein assay system.

Glucuronoxylan glucuronosyltransferase activity of microsomal proteins was assayed in a 100 μl reaction mixture with 100 mM NaAc, pH 7.0, 100 μg microsomal proteins, 1 mM UDP-GlcA (Sigma), 2 mM xylohexaose (Megazyme) and 5 mM MnCl_2_. For GX methyltransferase activity assay, the reaction mixture was added with 1 mM S-(5′-adenosyl)-L-methionine chloride dihydrochloride (Sigma) and 1 mM CoCl_2_. After incubation at 23°C for 3 h, the reaction mixtures were passed through a cut-off ultrafiltration tube (3 KD, Millipore). The percolate was directly subjected to LC-QTOF-MS for product identification. Percolate of GX glucuronosyltransferase activity assay mixture was stored at -20°C for the following methyltransferase assays.

### Methyltransferase Assay of Recombinant *PtrDUF579-3* and *PtrDUF579-9* Proteins

The DUF579 domain sequence of *PtrDUF579-3* and *PtrDUF579-9* was amplified using specific primer (Supplementary Table S1). The PCR products were sequenced and then cloned into the *NheI* and *SalI* sites of pET28b (Novagen). Recombinant proteins were induced in *Escherichia coli BL21*(*DE3*), purified with Ni-NTA Superflow (Qiagen) and examined by SDS-PAGE and Western blot using produced antibody according to the protocol (Song et al., 2010, 2014). Methyltransferase activity of the recombinant *PtrDUF579-3* and *PtrDUF579* proteins were analyzed in above 100 μl percolate containing glucuronxylohexaose by adding 100 μg recombinant *PtrDUF579-3* protein or 100 μg cell lysates of *E. coli BL21*(*DE3*) as control, 1 mM SAM and 1 mM CoCl_2_. After incubation at 23°C for 3 h, the reaction mixtures were passed through a cut-off ultrafiltration tube (3 KD, Millipore) and the percolate was directly subjected to LC-QTOF-MS for product identification.

### Liquid Chromatography-Quadrupole-Time-of-Flight-Mass Spectrometry

The product of methyltransferase assay were directly identified through LC-QTOF MS analysis using an Agilent 6520 series LC 1200 MS 6520 QTOF system packed with a ZORBAX Extend-C18 column (3.0 mm × 50 mm, 1.8 μm, Agilent, Palo Alto, CA, USA) as our previous protocol (Zhao et al., 2013). Briefly, 3 μl of xylo-oligosaccharides was injected with a constant mobile phase flow rate of 0.3 ml min^-1^. The mobile phase consisted of 10 mM ammonium acetate in H_2_O (A) and 20 mM ammonium acetate in acetonitrile (B) using a gradient elution of buffer B. GX oligosaccharides (1 μg/μl) extracted from wild type and transgenic plants were separated by XBRIDGE amide column (4.6 mm × 250 mm, 3.5 μm Waters, Milford, MA, USA) for structure characterization. The mobile phase consisted of 0.2% NH_4_OH in H_2_O (A) and pure acetonitrile (B) using a gradient elution of buffer B by a linear decrease from 80 to 50% in 30 min. The TOF mass spectrometer was set as scan range from 200 to 1700 at 160 V and radiofrequency (Sanderfoot et al., 1998) at 750 V in positive scan mode at 4 GHz resolution. The temperature of dry gas of electrospray ionization (ESI) was set at 350°C with holding flow at 9 L min^-1^.

### ^1^H-NMR Spectroscopy

Xylo-oligosaccharides used for NMR analysis were prepared from wild type plants or five plants of individual transgenic lines. Xylo-oligosaccharides samples (20 mg) were prepared with 100% D_2_O (0.6 mL, 99.9 atom % D; Sigma) in 5 mm standard NMR tubes. A total of 0.5 μl of acetone (δ 2.225) was added for measurement of chemical shifts. High-resolution NMR spectra were acquired at 303 K with a Varian Inova-NMR spectrometer (Varian, Palo Alto, CA, USA) operating at 600 MHz using a 5-mm NMR cold probe (Agilent). For all examination, 128 transients were collected and an acquisition time of 3-s. Residual water resonance was suppressed using the NOESYPR1D (1D Nuclear Overhauser effect spectroscopy with water pre-saturation) pulse sequence with a mixing time of 100 ms. 1D spectra were phased, baseline corrected for integral analysis using MestRe-Nova software (MestreC Research). All NMR spectra were compared with the spectra data for xylan structure analysis (Pena et al., 2007; Lee et al., 2011).

### GX Release and Cellulose Hydrolysis Assay

For GX release assay, wood samples collected from 1-year-old trees were milled to pass the 80 mesh screen for preparing AIRs according to (Foster et al., 2010). After de-starched procedure and iodine test, 2 mg of wood mill per reaction was weighted into a 2 mL screw cap tube with 300 μL 2% sulfuric acid, 20 ul of inositol (1 μg/μl) was used as an internal standard. After set at room temperature for 20 min, the reactions were heated to 121°C for 15, 25, 35, 45, 55 min. The reactions were immediately cooled down on ice after heated with various time intervals and neutralized with ammonium hydroxide. After centrifugation, 200 ul of supernatant was collected and dried in a rotatory evaporator and subjected to alditol acetate derivatization followed by acetylation procedure according to (Foster et al., 2010). Xylose content was detected and quantified using an Agilent GC–MS system equipped with a SP-2380 capillary column (Foster et al., 2010).

For 60 min acid treatment, 0.5 g of wood residue was collected by centrifugation and washed, and then suspended in 10 mL of 0.1 M sodium citrate buffer at pH 4.8. Cellulase (E.C. 3.2.1.4) from *T. viride* (Yakult Honsha, Tokyo, Japan) of 20 filter paper units (FPUs) was added to each sample and the volume was adjusted to 30 mL for digestion at 50°C for 1, 8, and 72 h. The glucose concentration in the reaction was measured by dinitrosalicylic acid (DNS) method using glucose as standard according to (Hou and Li, 2011).

## Results

### Catalytic Activity of GX Glucuronosyltransferases and Methyltransferase in *Populus* Developing Xylem Tissue

**FIGURE 1:**
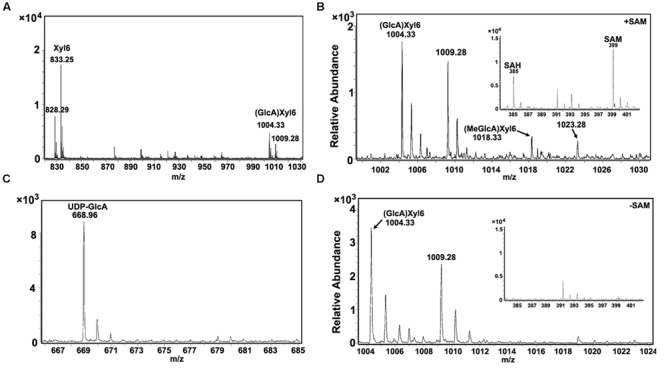
**Enzyme activity analysis of *Populus* xylem microsomal proteins. (A)** Using xylohexaose (Xyl_6_) and UDP-GlcA as substrates, glucuronosyltransferase activity in microsomal proteins was examined and the products were analyzed by LC-QTOF-MS. Xyl_6_ was identified with signal peaks of m/z [M+NH_4_]^+^ = 828.29 and m/z [M+Na]^+^ = 833.25. Product (GlcA)Xyl_6_ was identified with signal peaks of m/z [M+NH_4_]^+^ = 1004.33 and m/z [M+Na]^+^ = 1009.28. **(B)** Using xylohexaose, UDP-GlcA and SAM as substrates, GX methyltransferase activity in microsomal proteins was examined. Product (MeGlcA)Xyl_6_ was identified with signal peaks of m/z [M+NH_4_]^+^ = 1018.33 and m/z [M+Na]^+^ = 1023.28. The SAM and produced SAH signal peaks were at 399 and 385, respectively. **(C)** The UDP-GlcA was detected with signal peak of m/z [M+Na]^+^ = 668.96, while the UDP-GlcA methylated products (m/z [M+Na]^+^ = 682.96) was not detected, indicating that UDP-GlcA itself cannot be methylated in the reaction of b. **(D)** No (MeGlcA)Xyl_6_ was detected at the same reaction condition with absence of SAM. SAM, *S*-adenosyl methionine; SAH, *S*-adenosyl homocysteine.

To examine the GX methylation process during wood formation, the microsomal proteins from developing xylem tissue of *Populus* was isolated and determined for enzyme activities. Using UDP-GlcA and xylohexaose as substrates, glucuronoxylohexaose (GlcA)Xyl_6_ (m/z [M+NH_4_]^+^ = 1004.33; m/z [M+Na]^+^ = 1009.28; **Figure 1A**) was detected as a reaction product, indicating that the microsomal proteins contained a catalytic activity of glucuronosyltransferase. When UDP-GlcA, xylohexaose and *S*-adenosyl methionine (SAM) were used as substrates, both GlcAXyl_6_ and methyl-glucuronoxylohexaose (MeGlcA)Xyl_6_ (m/z [M+NH_4_]^+^ = 1018.33; m/z [M+Na]^+^ = 1023.28) was produced (**Figure 1B**), while the (MeGlcA)Xyl_6_ product was not be detected in the absence of SAM (**Figure 1D**), indicating the microsomal proteins have methylation activity on glucuronoxylohexaose using SAM as a methyl group donor. In this reaction, methylated UDP-GlcA was not found to be a product (**Figure 1C**), suggesting that UDP-GlcA may not be directly methylated in *Populus* xylem tissue, instead that the methylation may occur after the GlcA group is added onto GX polymers. These results revealed that the developing xylem microsomal protein contains catalytic activities for transferring the GlcA residues onto GX polymers as well as for catalyzing the methylation on the GlcA side chains.

**FIGURE 2:**
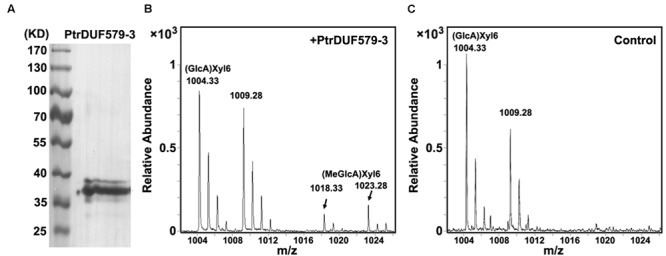
**Enzyme activity analysis of recombinant PtrDUF579-3 protein. (A)** Purified recombinant *PtrDUF579-3* proteins examined by Western blot. **(B)** GX methyltransferase activity of the recombinant *PtrDUF579-3* proteins was analyzed using (GlcA)Xyl_6_ as substrate. Product (MeGlcA)Xyl_6_ was identified from the *PtrDUF579-3* catalysis **(C)** (MeGlcA)Xyl_6_ was not detected from the *Escherichia coli* cell lysates catalysis.

**FIGURE 3:**
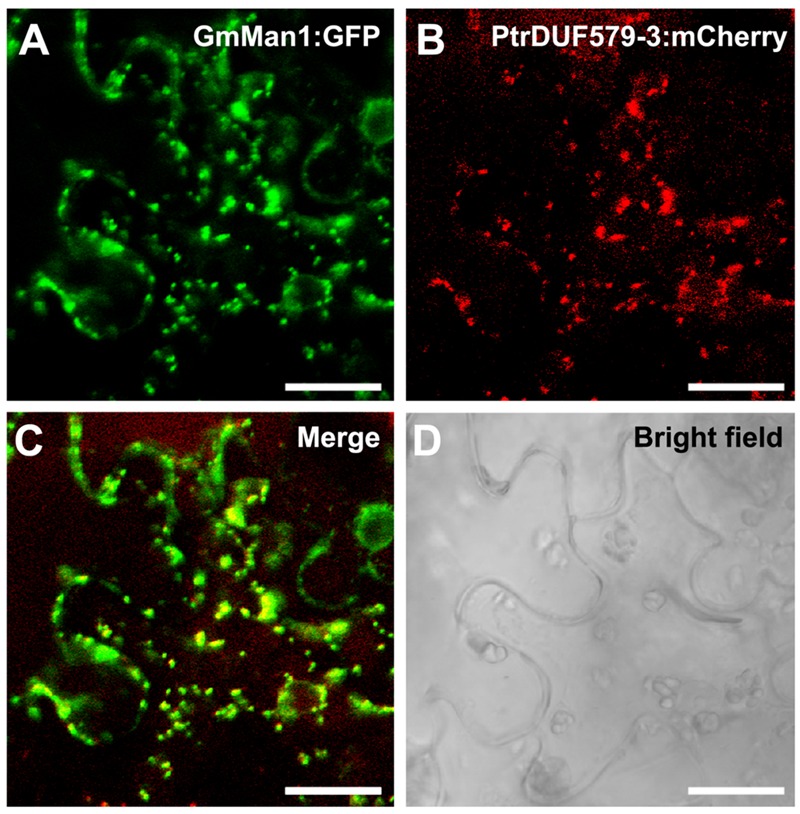
**Subcellular localization of PtrDUF579-3.** *PtrDUF579-3* protein was labeled with mCherry and its co-localization with Golgi marker, GmMan1:GFP. The signal of *PtrDUF579-3*:mCherry matched with GmMan1:GFP **(A–D)**. Bars: 20 μm.

In our previous study, *PtrDUF579-3* and *PtrDUF579-9* were identified for their high expression in developing xylem (Song et al., 2014). To examine *PtrDUF579-3* and *PtrDUF579-9* catalytic activity, their recombinant proteins were expressed in *E. coli* and purified (**Figure 2A**, **Supplementary Figures S1A,C**). The recombinant proteins were examined with their enzymatic activity in the reaction system containing GlcA-Xyl_6_ and SAM (methyl group donor) as substrates. *PtrDUF579-3* displayed a catalytic activity in producing MeGlcA-Xyl_6_ (**Figures 2B,C**). However, *PtrDUF579-9* was unable to catalyze the MeGlcA-Xyl_6_ production (**Supplementary Figure S1B**), indicating that *PtrDUF579-9* was deficient in such methylation under the experimental conditions. These suggest that the *PtrDUF579-3* but not *PtrDUF579-9* was able to methylate glucuronoxylan. *PtrDUF579-3* protein was further examined for its subcellular location. The Golgi apparatus markers GmMan1 were co-expressed with *PtrDUF579-3*:mCherry in tobacco leaf. *PtrDUF579-3*:mCherry shows colocalization with the Golgi apparatus markers (**Figures 3A–D**). The results indicated *PtrDUF579-3* localized in Golgi apparatus and mediated GX methylation process in *Populus* xylem cell.

### Suppression of *PtrDUF579-3* Affected the GX Components

**FIGURE 4:**
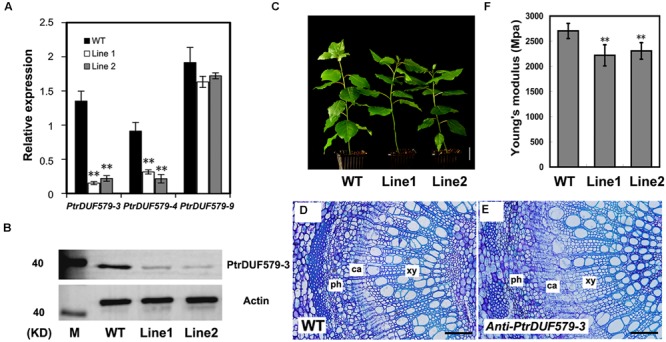
**Analysis of the *PtrDUF579-3* suppressed plants. (A)** Expression of *PtrDUF579-3, PtrDUF579-4*, and *PtrDUF579-9* in transgenic (Line 1 and Line 2) and wild type (WT) plants using *PtrActin2* as reference gene. The values are mean ± SE. *n* = 5. **(B)** *PtrDUF579-3* protein was dramatically reduced in the transgenic plants. **(C)** Morphology of 2 months-old *anti-PtrDUF579-3* transgenic (Line 1 and Line 2) plants. Bars: 5 cm. Cross-sections show the secondary vascular tissues in the 12th internode of WT plants **(D)** and transgenic plants **(E)** of 2 months-old. Bars: 200 μm. **(F)** Mechanical strength was decreased in *PtrDUF579-3* suppressed plants. Each transgenic line was multiplied through cutting propagation for analysis of biological repeats. The values are mean ± SE. *n* = 15. Significance as determined by Student’s *t*-test. ^∗∗^*P* < 0.01.

To investigate the *PtrDUF579-3* function *in planta*, an antisense *PtrDUF579-3* construct was transferred into *Populus* to generate *PtrDUF579-3* suppressed plants. Thirty-four independent transgenic lines were generated and confirmed. Expression of *PtrDUF579-3* was found to be down-regulated in twenty of the transgenic lines, among which two transgenic lines (Line 1 and line 2) were multiplied through cutting propagation and used for detailed analysis (**Figure 4A**). In the transgenic plants, expression of *PtrDUF579-3* and *PtrDUF579-4* was found to be simultaneously down-regulated, while the expression of *PtrDUF579*-*9* gene was not affected (**Figure 4A**). Western blot analysis demonstrated that *PtrDUF579-3* protein was dramatically reduced in the xylem tissue of transgenic plants (**Figure 4B**). Results also showed that both *PtrDUF579-3* and *PtrDUF579-4* share high protein sequence identity and same subcellular localization (**Supplementary Figure S2**). Likely *PtrDUF579-3* and *PtrDUF579-4* may play redundant roles. The transgenic plants did not show apparent growth alterations in terms of plant height, stem diameter, internode length, and leaf size compared to those in wild type plants (**Figure 4C**). Cross-section of the transgenic plant stem showed that the xylem cell morphology was not affected when observed under light microscope (**Figures 4D,E**). However, the mechanical strength of the stem was slightly reduced in the transgenic plants (**Figure 4F**). Changes of the stem mechanical property could be due to the alteration occurred in wood tissues.

The chemical composition of the wood was analyzed in transgenic plants. Compared to control plants, GlcA content was significantly decreased, while other monosaccharide composition was generally not changed in the transgenic plants, except a slight decrease in mannose content (**Table 1**). Furthermore, cellulose content and Klason lignin content were not significantly changed in the transgenic plants. Lignin S/G ratio was also not changed (**Table 1**). These results suggest suppression of *PtrDUF579-3* did not affect biosynthesis of cellulose and lignin but mainly affected the GlcA content in xylem cell walls.

**Table 1:** Chemical composition in the cell walls of xylem tissues.

Plant	Rha	Ara	Xyl	Man	Gal	Glc	GalA	GlcA	Cellulose	Klason Lignin	S/G Ratio
WT	0.65 ± 0.2	3.2 ± 1.1	70.23 ± 5.8	7.27 ± 1.1	5.63 ± 0.6	5.9 ± 1.8	8.92 ± 1.4	1.18 ± 0.2	45.20 ± 0.91	24.50 ± 0.81	2.28 ± 0.15
Line 1	0.98 ± 0.3	3.97 ± 1.1	67.33 ± 2.3	5.23 ± 0.9^∗^	5.08 ± 0.7	5.65 ± 0.9	8.55 ± 2.3	0.47 ± 0.1^∗∗^	45.42 ± 0.92	24.80 ± 0.79	2.35 ± 0.11
Line 2	0.87 ± 0.2	4.58 ± 0.8	68.54 ± 3.3	5.52 ± 0.4^∗^	4.78 ± 1.3	4.82 ± 1.6	10.42 ± 3.9	0.54 ± 0.1^∗∗^	44.64 ± 0.87	23.65 ± 0.92	2.20 ± 0.16

*WT, Wild-type plants; Line 1, Line 2, *Anti*-*PtrDUF579-3* transgenic lines. Rha, rhamnose; Fuc, fucose; Ara, arabinose; Xyl, xylose; Man, mannose; Gal, galactose; Glc, glucose; GalA, galacturonic acid; GlcA, total glucuronic acid. Monosaccharides, Mol % in alcohol-insoluble residues; cellulose and lignin, g/100 g dry weight. Significance as determined by Student’s *t*-test: ^∗∗^*P* < 0.01, ^∗^*P* < 0.05. Five trees per line were used in the assays. Values are mean ± SE of three independent assays.*

**FIGURE 5:**
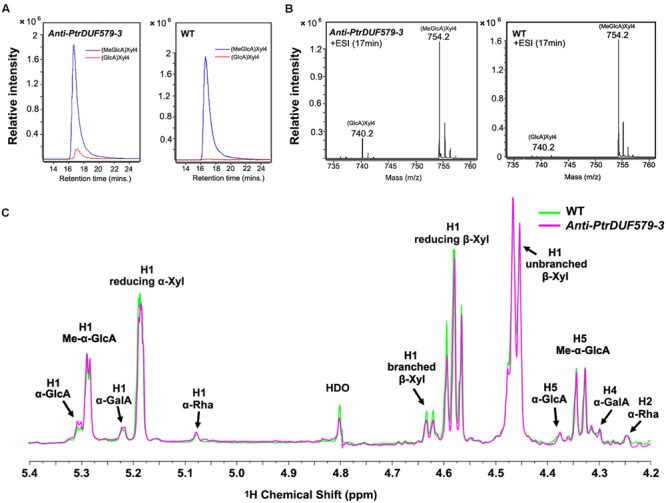
**Structure analysis of xylo-oligosaccharides by LC-QTOF-MS and NMR spectroscopy. (A)** Extracted ion chromatograph of MeGlcA-Xyl_4_ and GlcA-Xyl_4_ in *anti-PtrDUF579-3* transgenic plants (Line 1) and wild type (WT) plants. The abundance of GlcA-Xyl_4_ was elevated in the transgenic plants compared to that of WT plants. **(B)** Mass spectrometric detection of GX oligosaccharides MeGlcA-Xyl_4_ (m/z [M+NH4]^+^ = 754.2) and GlcA-Xyl_4_ (m/z [M+NH4]^+^ = 740.2). **(C)** The abundance of branched β-Xyl was decreased in *anti-PtrDUF579-3* transgenic plants (Line 1) indicating the reduction of GlcA side chains compared to that of WT examined by NMR. The abundance of α-GalA and α-Rha in reducing end tetrasaccharide was not changed in *anti-PtrDUF579-3* transgenic plants.

*PtrDUF579-3* protein displayed a catalytic activity of GX methyltransferase. Then we examined the methylation of the GX polymer in *Populus* wood cell walls. After GX was isolated and digested by endoxylanase, the released oligosaccharides were analyzed using a liquid chromatography quadrupole time-of-flight mass spectrometry (LC-QTOF-MS). The detected oligosaccharides from wild type *Populus* contained abundant MeGlcA-Xyl_4_ along with trivial GlcA-Xyl_4_ (**Figures 5A,B**). However, the GlcA-Xyl_4_ abundance was increased in the transgenic plants compared to wild type (**Figures 5A,B**), indicating a reduction of GlcA-Xyl_4_ methylation in the *PtrDUF579-3*-suppressed transgenics.

The GX structures in the transgenic plants were further examined by nuclear magnetic resonance (NMR). GX oligosaccharides from wild *Populus* displayed abundant MeGlcA residues with minor GlcA residues (**Figure 5C**). In the transgenic plant, un-methylated GlcA side chains on GX oligosaccharides were increased, while the methylated GlcA side chains were relatively reduced (**Figure 5C**; **Table 2**). This further verifies that *PtrDUF579-3* suppression resulted in reduction of the GlcA side chain methylation. Meanwhile, in GX polymers, the branched β-Xyl residues were reduced about 12% suggesting a reduction of GlcA/MeGlcA branches on the xylan backbone (**Table 2**). These results together suggest that *PtrDUF579-3* suppression led to reduction of GlcA methylation and branches on GX backbone. In addition, the α-GalA and α-Rha at GX reducing ends were not changed in the transgenic plant (**Figure 5C**; **Table 2**). As tetrasaccharide at the reducing ends can be referred to be correlated with the average degree of polymerization of GX (Pena et al., 2007), this indicated that the elongation of GX backbone was not affect in the *PtrDUF579-3* suppressed plants.

**Table 2:** Characterization of GX structure in *PtrDUF579-3* suppressed plants by NMR.

Plant	Non-methylated GlcA (%)	Methylated GlcA(%)	Relative branched β-Xyl	Relative reducing ends
WT	3	97	100	100
Line 1	7	93	88	98
Line 2	8	92	86	99

*WT, wild-type plants; Line 1, Line 2, *Anti*-*PtrDUF579-3* transgenic lines. Non-methylated GlcA: GlcA/total GlcA × 100%. Methylated GlcA: MeGlcA/total GlcA × 100%. Relative branched β-Xyl: the ratio of branched β-Xyl/total xylose in comparison with wild type. Relative reducing ends: the ratio of α-GalA+ β-Xyl and reducing ends are set as 100% in wild-type GX.*

### Analysis of Polysaccharide Hydrolysis of Wood Tissue

**FIGURE 6:**
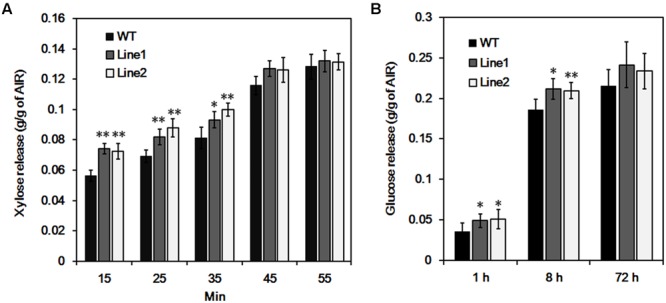
**Hydrolysis analysis of wood tissue. (A)** Xylose release from wood in the process of acid pretreatment. **(B)** Glucose release by cellulase hydrolysis after pretreatment. Each transgenic line was multiplied through cutting propagation for analysis of biological repeats. WT, wild type plants; Line 1, Line 2, *anti-PtrDUF579-3* transgenic lines. The values are mean ± SE. *n* = 5. Significance was determined by Student’s *t*-test of values of wild type and *PtrDUF579-3* suppressed plants. ^∗∗^*P* < 0.01; ^∗^*P* < 0.05.

Glucuronoxylan is involved in connecting lignin and cellulose fibrils together in wood and is thought to be a main factor limiting the hydrolytic efficiency during biomass conversion (Laureano-Perez et al., 2005; Himmel et al., 2007). We examined GX hydrolysis during acid treatment and cellulose digestibility after the treatment. The xylose release was significantly enhanced (average 29.9% in 15 min, 22.4% in 25 min, and 18.9% in 35 min) from *PtrDUF579-3* suppressed plants compared to the wild type plants during the course of treatment, but not significant after 45 min (**Figure 6A**). After treatment for 60 min, wood residues were subjected to cellulose digestibility assay. The glucose release was increased (average 38.3% in 1 h and 13.5% in 8 h) from *PtrDUF579-3* suppressed plants compared to the wild type plants with cellulase hydrolysis (**Figure 6B**). The results revealed that the *PtrDUF579-3* suppression enhances GX susceptibility to acid treatment and increased cellulose digestibility, which could attribute to the alteration of GX structure in the *PtrDUF579-3* suppressed plants.

## Discussion

In *Populus*, 12 *DUF579* genes have been identified, of which individual members play diverse roles in various developing processes (Song et al., 2014). In this study, evidence showed that *PtrDUF579-3* functions with an enzymatic activity in catalyzing methylation of GX side chains and plays a role *in planta* for affecting GX biosynthesis during wood formation.

### Distinct Enzyme Activities of DUF579 Proteins from *Populus*

Three DUF579 proteins from *Arabidopsis* have been studied for their biochemical function as SAM-dependent polysaccharide *O*-methyl transferase (OMT; Lee et al., 2012b; Urbanowicz et al., 2012). GXM1, GXM2, and GXMT1(GXM3) were able to catalyze the methylation of GlcA groups on GX polymer (Lee et al., 2012b; Urbanowicz et al., 2012). Knockout of *GXM* genes decrease the degree of GX methylation in *Arabidopsis*. However, knockout of other group DUF579 genes, *IRX15* and *IRX15L*, caused an increase of GX methylation. In this study, two recombinant proteins from *Populus DUF579* genes, *PtrDUF579-3* (homolog of *GXMT1*) and *PtrDUF579-9* (homolog of *IRX15*), displayed different enzyme activities. *PtrDUF579-3* is able to catalyze the methylation of GX oligosaccharides, whereas *PtrDUF579-9* did not show such activity in the same condition. In another study, *PtrDUF579-1* protein was reported to display GX methyltransferase activity, while its substrate affinity and catalytic efficiency were rather lower than those of *PtrDUF579-3* (Yuan et al., 2014). In our previous study, *PtrDUF579-1* was found to be localized in cambium cells. Suppression of *PtrDUF579-1* expression affected cambium pectin composition, suggesting its role in association with pectin formation in primary cell wall (Song et al., 2014). Here, the evidence suggests a possibility that DUF579 family members may have different catalyzing properties or catalyze methylation using different polysaccharide substrates. Certainly this remains to be further investigated.

### Roles of PtrDUF579-3 in the GX Biosynthesis Process

In *Arabidopsis* (Columbia type), about 63% of the GlcA side chains on the GX backbone is methylated (Urbanowicz et al., 2012). In our study, about 97% of the GlcA side chains in *Populus* xylem tissue are found to be methylated. The different degree of GlcA side chain methylation between *Arabidopsis* and *Populus* may indicate a difference of GX polymer structures in the two plants.

Activity analysis indicated that the *Populus* microsomal proteins contain glucuronosyltransferases and methyltransferases. Recombinant *PtrDUF579-3* protein displayed GX methyltransferase activity. Consistent with this, genetic suppression of *PtrDUF579-3* caused the GlcA methylation reduction of GX polymers *in planta*. In addition, branches on GX polymers were also decreased in the *PtrDUF579-3* suppressed plants. In *Arabidopsis*, knocking-out of a *DUF579* gene, *GXMT1* affected the methylation of GlcA, but did not affect the addition of GlcA onto GX backbone (Lee et al., 2012b; Urbanowicz et al., 2012). A recent study shows that *PtrDUF579-3* could only restore a small fraction of GlcA side chains of GX in the *gxm1/2/3* mutant suggesting a possibility that GlcA methylation is carried out by a protein complex containing DUF579s and other xylan biosynthetic enzymes (Yuan et al., 2014). Thus, how GlcA methylation and its addition onto GX are carried out in a coordinated manner remains to be further characterized in *Populus*.

*PtrDUF579-3* and *PtrDUF579-4* share 90% sequence identity, with a similar expression and same subcellular localization. Suppression of *PtrDUF579-3* affected *PtrDUF579-4* but not other family members. Due to the difficulty in individual regulation of the two genes, the phenotypes of the *PtrDUF579-3*-suppressed plants could be due to simultaneous suppression of both *PtrDUF579-3* and *PtrDUF579-4* expression. Actually *PtrDUF579-3* and *PtrDUF579-4* form a pair of *DUF579* genes (Song et al., 2014) with similar enzyme activity (Yuan et al., 2014). Likely, they may play redundant roles.

### GX Structure and Wood Formation

As a potential feedstock for biofuel industry, wood is mainly composed of three types of biopolymers, cellulose, hemicellulose and lignin. Among these polymers, cellulose and lignin are connected by hemicelluloses in wood secondary cell walls (Fengel and Wegener, 1983). In hard wood species, GX is a major hemicellulose and affects conversion of wood biomass into biofuel (Laureano-Perez et al., 2005; Himmel et al., 2007; Lee et al., 2011). Engineering of the GX structure through genetically manipulating genes involved in GX biosynthesis may contribute to improvement of the wood conversion efficiency for biofuel production. In *Populus*, alteration of the GX content and structure through down-regulation of *GT43B* and *GAUT12* gene enhanced sugar release from wood biomass (Lee et al., 2011; Biswal et al., 2015). In this study, suppression of a glucuronoxylan methyltransferase gene, *PtrDUF579-3*, enhances GX susceptibility to acid treatment which would be beneficial for pentose separation in the process of pretreatment.

In secondary walls, it is thought that GX interacts with cellulose and lignin. The backbone of GX interacts with cellulose microfibrils through hydrogen-bond (Laureano-Perez et al., 2005), while the substituted GX with negatively charged MeGlcA side residues has low affinity with cellulose (Linder et al., 2003; Kabel et al., 2007). The GlcA substitutions may allow xylan backbone to fold as a twofold helical screw to form an uncharged surface to facilitate GX-cellulose interaction or to fold as threefold helical screw to form a charged surface for interaction of GlcA residue with other cell wall components (Szabo et al., 2001; Bromley et al., 2013). The twofold helical screw conformation allows the xylan to interact with both hydrophilic and hydrophobic faces of cellulose (Busse-Wicher et al., 2014). In addition, the MeGlcA side residues on GX polymers are reported to be able to interact with lignin through covalent ester linkages (Takahashi and Koshijima, 1988). Regulation of *PtrDUF579-3* in *Populus* caused GX methylation and substitution changes. The change of GX structure may affect the direct linkages between xylan, cellulose microfibrils, lignin, and other cell wall components. The enhanced cellulose accessibility by cellulase in the *PtrDUF579-3* suppressed plants could be caused by alternation of the linkage networks, suggesting that suppression of *PtrDUF579-3* can be a useful approach for wood property engineering, which would be beneficial for industrial biofuel production.

## Author Contributions

Conceived and designed the experiments: DS, LL. Performed the experiments: DS, JG, CL, JS. Analyzed the data: DS, LL. Wrote the paper: DS, LL. All the authors have read and approved the final manuscript.

## Conflict of Interest Statement

The authors declare that the research was conducted in the absence of any commercial or financial relationships that could be construed as a potential conflict of interest.

## Supplementary Material

FIGURE S1 **Enzyme activity analysis of recombinant PtrDUF579-9 protein. (A)** Purified recombinant *PtrDUF579-9* proteins were examined by Western blot. **(B)** GX methyltransferase activity of the recombinant *PtrDUF579-9* protein was analyzed. No (MeGlcA)Xyl_6_ was identified from the *PtrDUF579-9* catalysis. **(C)** Purified recombinant *PtrDUF579-3* and *PtrDUF579-9* proteins which were separated by sodium dodecyl sulfate polyacrylamide (SDS-PAGE) gel.

FIGURE S2 **Subcellular localization of PtrDUF579-4. (A)** *PtrDUF579-4* has high protein sequence identity with *PtrDUF579-3* analyzed by sequence alignment. The sequences of peptides used for *PtrDUF579-3* antibody production were underlined. **(B)** *PtrDUF579-4* protein was labeled with mCherry and its co-localization with Golgi marker, GmMan1:GFP. The signal of *PtrDUF579-4*:mCherry matched with GmMan1:GFP (A–H). Bars: 20 μm.

## References

[B1] Bauer S. Vasu P. Persson S. Mort A. J. Somerville C. R. (2006). Development and application of a suite of polysaccharide-degrading enzymes for analyzing plant cell walls. *Proc. Natl. Acad. Sci. U.S.A.* 103 11417–11422. 10.1073/pnas.0604632103 PMC1544100 16844780

[B2] Biswal A. K. Hao Z. Pattathil S. Yang X. Winkeler K. Collins C. (2015). Downregulation of GAUT12 in Populus deltoides by RNA silencing results in reduced recalcitrance, increased growth and reduced xylan and pectin in a woody biofuel feedstock. *Biotechnol. Biofuels* 8:41 10.1186/s13068-015-0218-y PMC4369864 25802552

[B3] Bromley J. R. Busse-Wicher M. Tryfona T. Mortimer J. C. Zhang Z. Brown D. (2013). GUX1 and GUX2 glucuronyltransferases decorate distinct domains of glucuronoxylan with different substitution patterns. *Plant J.* 74 423–434. 10.1111/tpj.12135 23373848

[B4] Brown D. Wightman R. Zhang Z. Gomez L. D. Atanassov I. Bukowski J. P. (2011). *Arabidopsis* genes IRREGULAR XYLEM (IRX15) and IRX15L encode DUF579-containing proteins that are essential for normal xylan deposition in the secondary cell wall. *Plant J.* 66 401–413. 10.1111/j.1365-313X.2011.04501.x 21251108

[B5] Brown D. M. Goubet F. Wong V. W. Goodacre R. Stephens E. Dupree P. (2007). Comparison of five xylan synthesis mutants reveals new insight into the mechanisms of xylan synthesis. *Plant J.* 52 1154–1168. 10.1111/j.1365-313X.2007.03307.x 17944810

[B6] Brown D. M. Zhang Z. Stephens E. Dupree P. Turner S. R. (2009). Characterization of IRX10 and IRX10-like reveals an essential role in glucuronoxylan biosynthesis in *Arabidopsis*. *Plant J.* 57 732–746. 10.1111/j.1365-313X.2008.03729.x 18980662

[B7] Busse-Wicher M. Gomes T. C. F. Tryfona T. Nikolovski N. Stott K. Grantham N. J. (2014). The pattern of xylan acetylation suggests xylan may interact with cellulose microfibrils as a twofold helical screw in the secondary plant cell wall of *Arabidopsis thaliana*. *Plant J.* 79 492–506. 10.1111/tpj.12575 24889696 PMC4140553

[B8] Chang S. Puryear J. Cairney J. (1993). A simple and efficient method for isolating RNA from pine trees. *Plant Mol. Biol. Rep.* 11 113–116. 10.1007/BF02670468

[B9] Fengel D. Wegener G. (1983). *Wood: Chemistry, Ultrastructure, Reactions*. Berlin: Walter de Gruyter 10.1515/9783110839654

[B10] Foster C. E. Martin T. M. Pauly M. (2010). Comprehensive compositional analysis of plant cell walls (lignocellulosic biomass) part II: carbohydrates. *JOVE J. Vis. Exp.* 37:e1745. 10.3791/1837 PMC3145335 20228730

[B11] Gui J. Shen J. Li L. (2011). Functional characterization of evolutionarily divergent 4-coumarate:coenzyme a ligases in rice. *Plant Physiol.* 157 574–586. 10.1104/pp.111.178301 21807887 PMC3192572

[B12] Himmel M. E. Ding S. Y. Johnson D. K. Adney W. S. Nimlos M. R. Brady J. W. (2007). Biomass recalcitrance: engineering plants and enzymes for biofuels production. *Science* 315 804–807. 10.1126/science.1137016 17289988

[B13] Hou S. Li L. (2011). Rapid characterization of woody biomass digestibility and chemical composition using near infrared spectroscopy. *J. Integr. Plant Biol.* 53 166–175. 10.1111/j.1744-7909.2010.01003.x 21261813

[B14] Jensen J. K. Kim H. Cocuron J. C. Orler R. Ralph J. Wilkerson C. G. (2011). The DUF579 domain containing proteins IRX15 and IRX15-L affect xylan synthesis in *Arabidopsis*. *Plant J.* 66 387–400. 10.1111/j.1365-313X.2010.04475.x 21288268

[B15] Johansson M. Samuelson O. (1977). Reducing end groups in brich xylan and their alkaline degradation. *Wood Sci. Technol.* 11 251–263. 10.1007/BF00356924

[B16] Kabel M. A. van den Borne H. Vincken J.-P. Voragen A. G. Schols H. A. (2007). Structural differences of xylans affect their interaction with cellulose. *Carbohyd. Polym.* 69 94–105. 10.1016/j.carbpol.2006.09.006

[B17] Laureano-Perez L. Teymouri F. Alizadeh H. Dale B. E. (2005). Understanding factors that limit enzymatic hydrolysis of biomass. *Appl. Biochem. Biotechnol.* 121 1081–1099. 10.1385/ABAB:124:1-3:1081 15930583

[B18] Lee C. Teng Q. Huang W. Zhong R. Ye Z. H. (2009a). Down-regulation of PoGT47C expression in poplar results in a reduced glucuronoxylan content and an increased wood digestibility by cellulase. *Plant Cell Physiol.* 50 1075–1089. 10.1093/pcp/pcp060 19395414

[B19] Lee C. Teng Q. Huang W. Zhong R. Ye Z. H. (2009b). The F8H glycosyltransferase is a functional paralog of FRA8 involved in glucuronoxylan biosynthesis in *Arabidopsis*. *Plant Cell Physiol.* 50 812–827. 10.1093/pcp/pcp025 19224953

[B20] Lee C. Teng Q. Huang W. Zhong R. Ye Z. H. (2009c). The poplar GT8E and GT8F glycosyltransferases are functional orthologs of *Arabidopsis* PARVUS involved in glucuronoxylan biosynthesis. *Plant Cell Physiol.* 50 1982–1987. 10.1093/pcp/pcp131 19789274

[B21] Lee C. Teng Q. Zhong R. Ye Z. H. (2011). Molecular dissection of xylan biosynthesis during wood formation in poplar. *Mol. Plant* 4 730–747. 10.1093/mp/ssr035 21596688

[B22] Lee C. Teng Q. Zhong R. Ye Z. H. (2012a). *Arabidopsis* GUX proteins are glucuronyltransferases responsible for the addition of glucuronic acid side chains onto xylan. *Plant Cell Physiol.* 53 1204–1216. 10.1093/pcp/pcs064 22537759

[B23] Lee C. Teng Q. Zhong R. Yuan Y. Haghighat M. Ye Z. H. (2012b). Three *Arabidopsis* DUF579 domain-containing GXM proteins are methyltransferases catalyzing 4-O-methylation of glucuronic acid on Xylan. *Plant Cell Physiol.* 53 1934–1949. 10.1093/pcp/pcs138 23045523

[B24] Lee C. Zhong R. Richardson E. A. Himmelsbach D. S. McPhail B. T. Ye Z. H. (2007a). The PARVUS gene is expressed in cells undergoing secondary wall thickening and is essential for glucuronoxylan biosynthesis. *Plant Cell Physiol.* 48 1659–1672. 10.1093/pcp/pcm155 17991630

[B25] Lee C. H. O’Neill M. A. Tsumuraya Y. Darvill A. G. Ye Z. H. (2007b). The irregular xylem9 mutant is deficient in xylan xylosyltransferase activity. *Plant Cell Physiol.* 48 1624–1634. 10.1093/pcp/pcm135 17938130

[B26] Li L. Zhou Y. Cheng X. Sun J. Marita J. M. Ralph J. (2003). Combinatorial modification of multiple lignin traits in trees through multigene cotransformation. *Proc. Natl. Acad. Sci. U.S.A.* 100 4939–4944. 10.1073/pnas.0831166100 12668766 PMC153659

[B27] Li M. Xiong G. Li R. Cui J. Tang D. Zhang B. (2009). Rice cellulose synthase-like D4 is essential for normal cell-wall biosynthesis and plant growth. *Plant J.* 60 1055–1069. 10.1111/j.1365-313X.2009.04022.x 19765235

[B28] Li Q. Min D. Wang J. P. Y. Peszlen I. Horvath L. Horvath B. (2011). Down-regulation of glycosyltransferase 8D genes in Populus trichocarpa caused reduced mechanical strength and xylan content in wood. *Tree Physiol.* 31 226–236. 10.1093/treephys/tpr008 21450982

[B29] Linder Å. Bergman R. Bodin A. Gatenholm P. (2003). Mechanism of assembly of xylan onto cellulose surfaces. *Langmuir* 19 5072–5077. 10.1021/la0341355

[B30] Lluveras-Tenorio A. Mazurek J. Restivo A. Colombini M. P. Bonaduce I. (2012). Analysis of plant gums and saccharide materials in paint samples: comparison of GC-MS analytical procedures and databases. *Chem. Cent. J.* 6 1–16. 10.1186/1752-153X-6-115 23050842 PMC3541984

[B31] Mazumder K. Peña M. J. O’Neill M. A. York W. S. (2012). Structural characterization of the heteroxylans from poplar and switchgrass. *Methods Mol. Biol.* 908 215–228. 10.1007/978-1-61779-956-3_19 22843402

[B32] Mishra S. Naik J. Patil Y. (2000). The compatibilising effect of maleic anhydride on swelling and mechanical properties of plant-fiber-reinforced novolac composites. *Compos. Sci. Technol.* 60 1729–1735. 10.1016/S0266-3538(00)00056-7

[B33] Mortimer J. C. Miles G. P. Brown D. M. Zhang Z. Segura M. P. Weimar T. (2010). Absence of branches from xylan in *Arabidopsis gux* mutants reveals potential for simplification of lignocellulosic biomass. *Proc. Natl. Acad. Sci. U.S.A.* 107 17409–17414. 10.1073/pnas.1005456107 20852069 PMC2951434

[B34] Nelson B. K. Cai X. Nebenfuhr A. (2007). A multicolored set of in vivo organelle markers for co-localization studies in *Arabidopsis* and other plants. *Plant J.* 51 1126–1136. 10.1111/j.1365-313X.2007.03212.x 17666025

[B35] Pena M. J. Zhong R. Zhou G. K. Richardson E. A. O’Neill M. A. Darvill A. G. (2007). *Arabidopsis* irregular xylem8 and irregular xylem9: implications for the complexity of glucuronoxylan biosynthesis. *Plant Cell* 19 549–563. 10.1105/tpc.106.049320 17322407 PMC1867335

[B36] Sanderfoot A. A. Ahmed S. U. Marty-Mazars D. Rapoport I. Kirchhausen T. Marty F. (1998). A putative vacuolar cargo receptor partially colocalizes with AtPEP12p on a prevacuolar compartment in *Arabidopsis* roots. *Proc. Natl. Acad. Sci. U.S.A.* 95 9920–9925. 10.1073/pnas.95.17.9920 PMC21437 9707576

[B37] Song D. Shen J. Li L. (2010). Characterization of cellulose synthase complexes in *Populus xylem* differentiation. *New Phytol.* 187 777–790. 10.1111/j.1469-8137.2010.03315.x 20546138

[B38] Song D. Sun J. Li L. (2014). Diverse roles of PtrDUF579 proteins in *Populus* and PtrDUF579-1 function in vascular cambium proliferation during secondary growth. *Plant Mol. Biol.* 85 601–602. 10.1007/s11103-014-0206-9 24899403

[B39] Suzuki S. Li L. Sun Y. H. Chiang V. L. (2006). The cellulose synthase gene superfamily and biochemical functions of xylem-specific cellulose synthase-like genes in *Populus trichocarpa*. *Plant Physiol.* 142 1233–1245. 10.1104/pp.106.086678 PMC1630762 16950861

[B40] Szabo L. Jamal S. Xie H. F. Charnock S. J. Bolam D. N. Gilbert H. J. (2001). Structure of a family 15 carbohydrate-binding module in complex with xylopentaose - Evidence that xylan binds in an approximate 3-fold helical conformation. *J. Biol. Chem.* 276 49061–49065. 10.1074/jbc.M109558200 11598143

[B41] Takahashi N. Koshijima T. (1988). Ester linkages between lignin and glucuronoxylan in a lignin-carbohydrate complex from beech (*Fagus crenata*) wood. *Wood Sci. Technol.* 22 231–241. 10.1007/BF00386018

[B42] Timell T. E. (1967). Recent progress in the chemistry of wood hemicelluloses. *Wood Sci. Technol.* 1 45–70. 10.1007/BF00592255

[B43] Urbanowicz B. R. Peña M. J. Ratnaparkhe S. Avci U. Backe J. Steet H. F. (2012). 4-O-methylation of glucuronic acid in *Arabidopsis glucuronoxylan* is catalyzed by a domain of unknown function family 579 protein. *Proc. Natl. Acad. Sci. U.S.A.* 109 14253–14258. 10.1073/pnas.1208097109 22893684 PMC3435161

[B44] Wu A. M. Hornblad E. Voxeur A. Gerber L. Rihouey C. Lerouge P. (2010). Analysis of the *Arabidopsis* IRX9/IRX9-L and IRX14/IRX14-L pairs of glycosyltransferase genes reveals critical contributions to biosynthesis of the hemicellulose glucuronoxylan. *Plant Physiol.* 153 542–554. 10.1104/pp.110.154971 20424005 PMC2879767

[B45] Wu A. M. Rihouey C. Seveno M. Hörnblad E. Singh S. K. Matsunaga T. (2009). The *Arabidopsis* IRX10 and IRX10-LIKE glycosyltransferases are critical for glucuronoxylan biosynthesis during secondary cell wall formation. *Plant J.* 57 718–731. 10.1111/j.1365-313X.2008.03724.x 18980649

[B46] Yuan Y. Teng Q. Zhong R. Ye Z.-H. (2014). Identification and biochemical characterization of four wood-associated glucuronoxylan methyltransferases in populus. *PLoS ONE* 9:e87370 10.1371/journal.pone.0087370 PMC3921138 24523868

[B47] Zhao Y. Song D. Sun J. Li L. (2013). Populus endo-beta-mannanase PtrMAN6 plays a role in coordinating cell wall remodeling with suppression of secondary wall thickening through generation of oligosaccharide signals. *Plant J.* 74 473–485. 10.1111/tpj.12137 23384057

[B48] Zhong R. Pena M. J. Zhou G. K. Nairn C. J. Wood-Jones A. Richardson E. A. (2005). *Arabidopsis* fragile fiber8, which encodes a putative glucuronyltransferase, is essential for normal secondary wall synthesis. *Plant Cell* 17 3390–3408. 10.1105/tpc.105.035501 16272433 PMC1315377

[B49] Zhou G. K. Zhong R. Himmelsbach D. S. McPhail B. T. Ye Z. H. (2007). Molecular characterization of PoGT8D and PoGT43B, two secondary wall-associated glycosyltransferases in poplar. *Plant Cell Physiol.* 48 689–699. 10.1093/pcp/pcm037 17379696

[B50] Zhou G. K. Zhong R. Richardson E. A. Morrison W. H. III Nairn C. J. Wood-Jones A. (2006). The poplar glycosyltransferase GT47C is functionally conserved with *Arabidopsis* Fragile Fiber8. *Plant Cell Physiol.* 47 1229–1240. 10.1093/pcp/pcj093 16887843

