# Supplementary figures and images for: Suppression of PtrDUF579-3 Expression Causes Structural Changes of the Glucuronoxylan in Populus
Source: Front Plant Sci. 2016 Apr 11;7:493. doi: 10.3389/fpls.2016.00493 (PMC4827005; doi:10.3389/fpls.2016.00493)

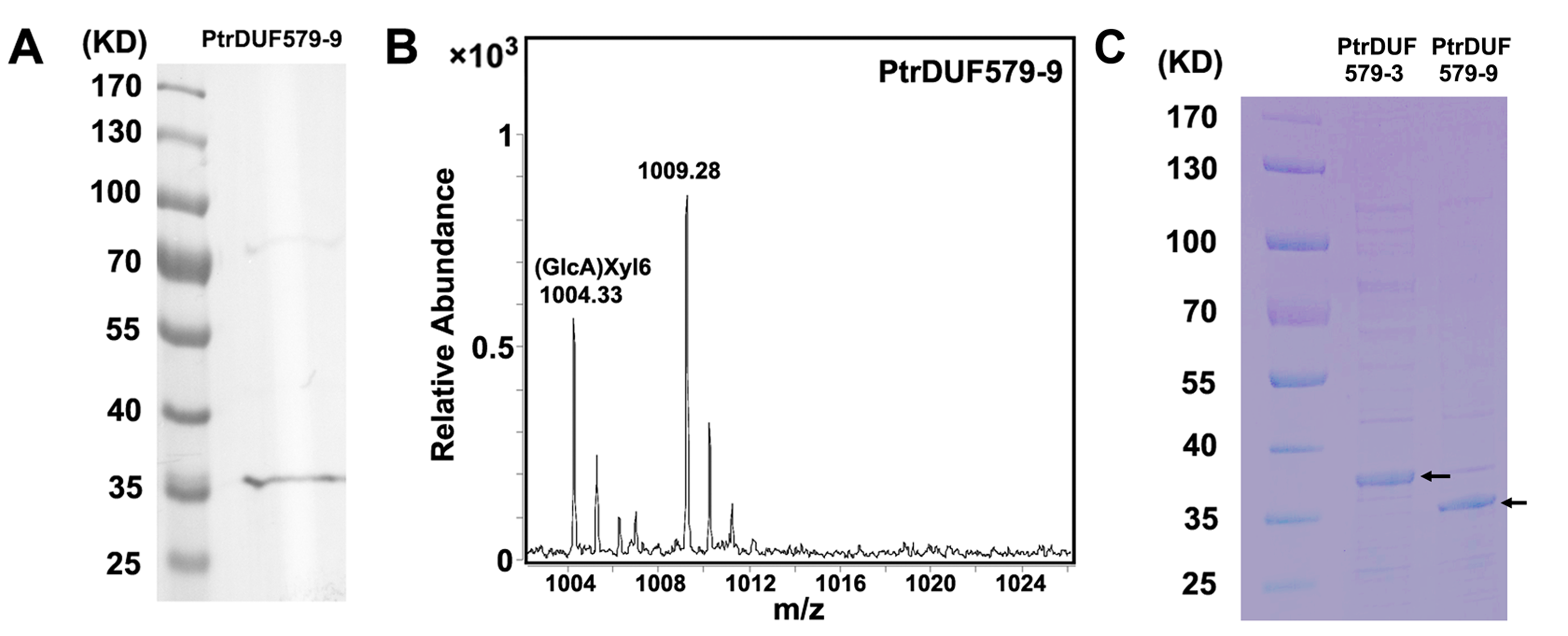

Supplement: FIGURE S1 — Enzyme activity analysis of recombinant PtrDUF579-9 protein. (A) Purified recombinant PtrDUF579-9 proteins were examined by Western blot. (B) GX methyltransferase activity of the recombinant PtrDUF579-9 protein was analyzed. No (MeGlcA)Xyl6 was identified from the PtrDUF579-9 catalysis. (C) Purified recombinant PtrDUF579-3 and PtrDUF579-9 proteins which were separated by sodium dodecyl sulfate polyacrylamide (SDS-PAGE) gel. [file Image_1.TIF]

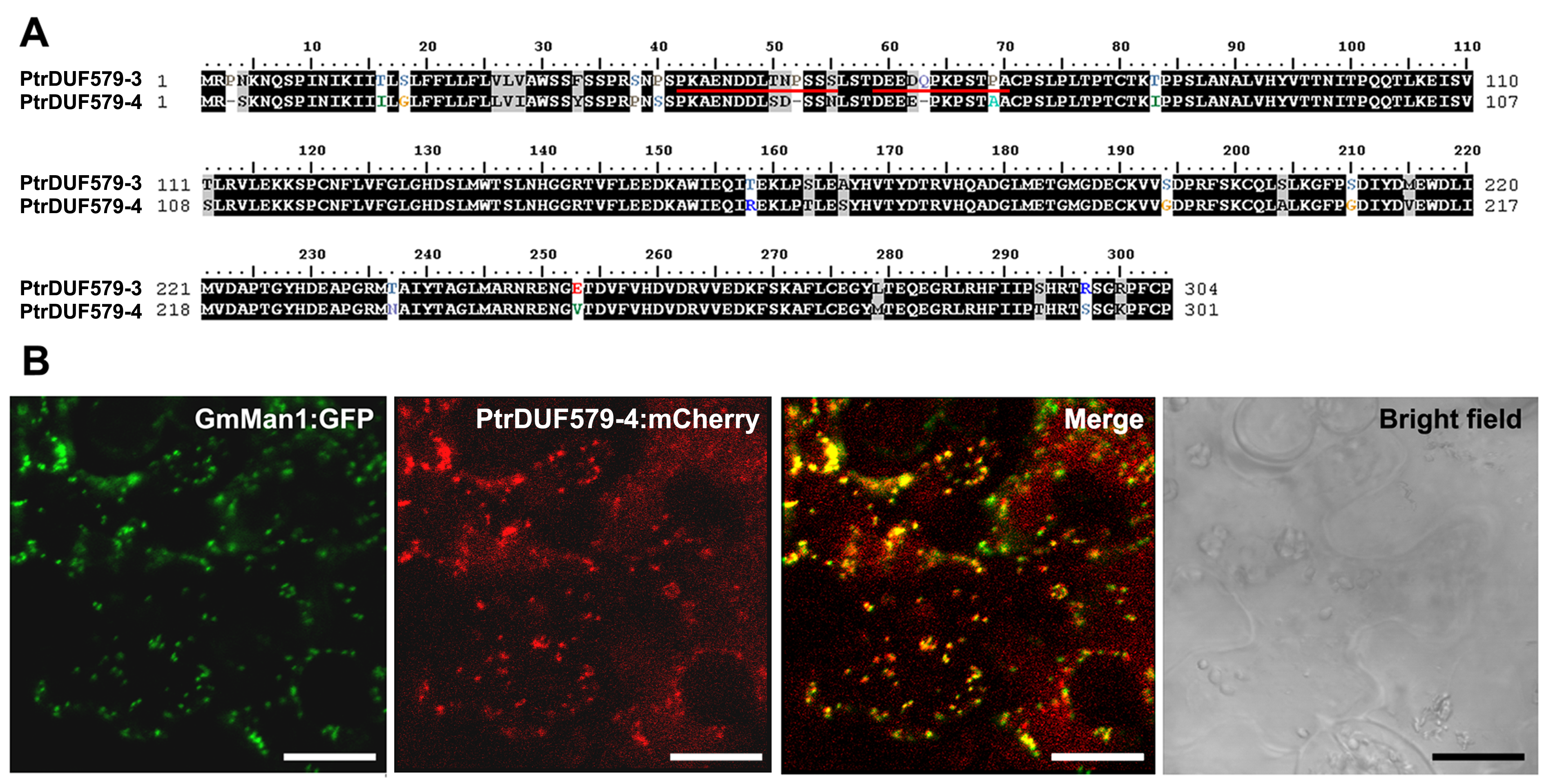

Supplement: FIGURE S2 — Subcellular localization of PtrDUF579-4. (A) PtrDUF579-4 has high protein sequence identity with PtrDUF579-3 analyzed by sequence alignment. The sequences of peptides used for PtrDUF579-3 antibody production were underlined. (B) PtrDUF579-4 protein was labeled with mCherry and its co-localization with Golgi marker, GmMan1:GFP. The signal of PtrDUF579-4:mCherry matched with GmMan1:GFP (A–H). Bars: 20 μm. [file Image_2.TIF]
